# Building Semipermeable Films One Monomer at a Time: Structural Advantages via Molecular Layer Deposition vs Interfacial Polymerization

**DOI:** 10.1021/acs.chemmater.3c02519

**Published:** 2024-01-18

**Authors:** Brian C. Welch, Emma N. Antonio, Thomas P. Chaney, Olivia M. McIntee, Joseph Strzalka, Victor M. Bright, Alan R. Greenberg, Tamar Segal-Peretz, Michael Toney, Steven M. George

**Affiliations:** † Israel Institute of Technology, Haifa 3200003, Israel; ‡ University of Colorado Boulder, Boulder, Colorado 80309, United States; § Argonne National Laboratory, Lemont, Illinois 60439, United States

## Abstract

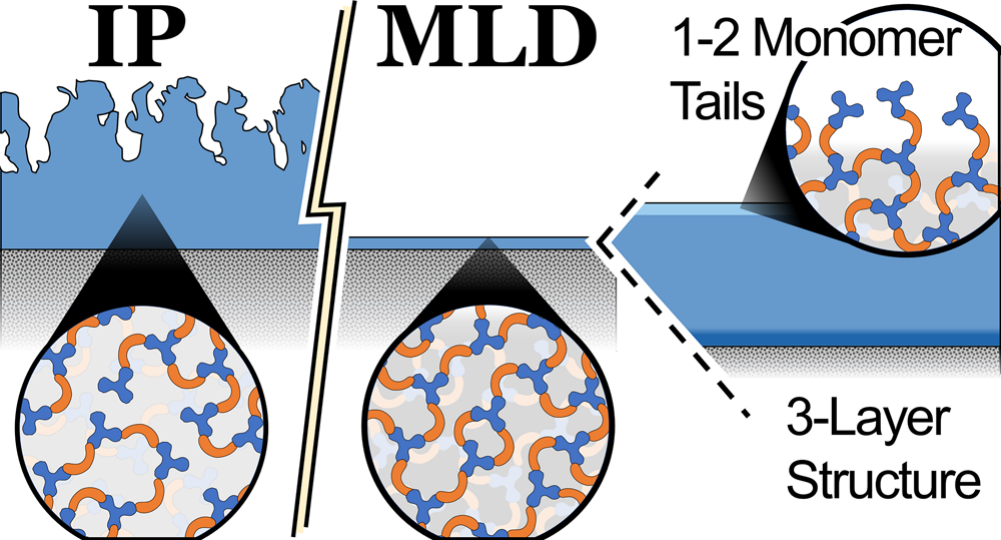

Molecular layer deposition (MLD) provides the opportunity to perform condensation polymerization one vaporized monomer at a time for the creation of precise, selective nanofilms for desalination membranes. Here, we compare the structure, chemistry, and morphology of two types of commercial interfacial polymerzation (IP) membranes with lab-made MLD films. M-phenylenediamine (MPD) and trimesoyl chloride (TMC) produced a cross-linked, aromatic polyamide often used in reverse osmosis membranes at MLD growth rates of 2.9 Å/cycle at 115 °C. Likewise, piperazine (PIP) and TMC formed polypiperazine amide, a common selective layer in nanofiltration membranes, with MLD growth rates of 1.5 Å/cycle at 115 °C. Ellipsometry and X-ray reflectivity results suggest that the surface of the MLD films is comprised of polymer segments roughly two monomers in length, which are connected at one end to the cross-linked bulk layer. As a result of this structure as well as the triple-functionality of TMC, MPD-TMC had a temperature window of stable growth rate from 115 to 150 °C, which is unlike any non-cross-linked MLD chemistries reported in the literature. Compared to IP films, corresponding MLD films were denser and morphologically conformal, which suggests a reduction in void volumes; this explains the high degree of salt rejection and reduced flux previously observed for exceptionally thin MPD-TMC MLD membranes. Using X-ray photoelectron spectroscopy and infrared spectroscopy, MLD PIP-TMC films evidenced a completely cross-linked internal structure, which lacked amine and carboxyl groups, pointing to a hydrophobic bulk structure, ideal for optimized water flux. Grazing-incidence wide-angle X-ray scattering showed broad features in each polyamide with *d*-spacings of 5.0 Å in PIP-TMC compared to that of 3.8 Å in MPD-TMC. While MLD and IP films were structurally identical to PIP-TMC, MPD-TMC IP films had a structure that may have been altered by post-treatment compared to MLD films. These results provide foundational insights into the MLD process, structure–performance relationships, and membrane fabrication.

## 1 Introduction

For decades, the industry standard for fabricating polyamide reverse osmosis (RO) and nanofiltration (NF) membranes has been interfacial polymerization (IP). A thin polymer film is synthesized on a support layer at the interface of two immiscible liquids. The diffusion–reaction mechanisms involved with IP are complex; thus, the ability to engineer films at the nanometer scale is limited.^1,2^ While commercially successful, IP membranes face shortcomings, which include inhomogeneous water passage, a propensity for fouling at the membrane surface, the extensive use of environmentally harmful solvents in synthesis, and a defect-prone manufacturing process that limits production rates.^3,4^

**Figure 1:**
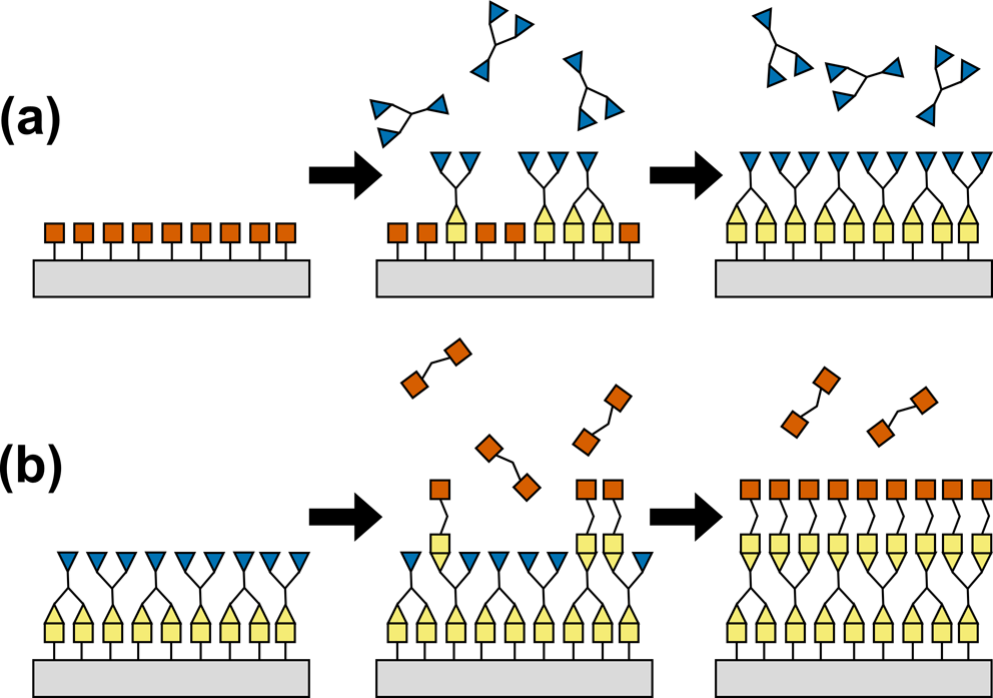
MLD process is composed of two steps: (a) functional groups on the substrate (squares) react with gas-phase precursors. Functional groups on the precursors (triangles) form a monolayer via covalent bonds with the surface. (b) The first precursor and byproducts are cleared away, and the substrate has new functionality for reaction with a second precursor. These two steps are cycled for continued film growth. Reproduced with permission from ref (8). Copyright 2021 Elsevier.

Molecular layer deposition (MLD) is a thin-film growth technique that has recently been explored for the fabrication of desalination membranes. MLD has untapped potential, capable of producing membrane films that are morphologically homogeneous, defect-free, and potentially antifouling without the use of solvents.^5−7^ Unlike IP, it provides control over the extent of growth at the single monomer level. The ultrathin yet conformal nature of MLD films is considered ideal for maximizing permeability while maintaining structural integrity. MLD is a derivative of atomic layer deposition (ALD) and shares its processing scheme: expose a substrate to monomer vapors one at a time as detailed in Figure 1. This sequential method is self-limiting at each exposure, which allows for precise film growth. However, MLD has nuances that deviate from ALD growth mechanisms. This work explores how these distinctions arise due to the macromolecular nature of organic MLD and how they affect the molecular structure of the synthesized films.

**Figure 2:**
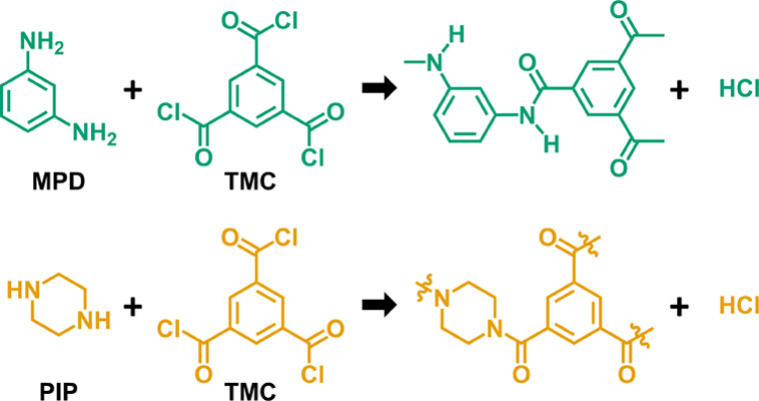
Polyamide condensation chemistries. M-phenylenediamine (MPD) and trimesoyl chloride (TMC) are used for reverse osmosis membranes. Piperazine (PIP) and TMC are used for nanofiltration membranes.

Two cross-linked, semipermeable polyamide MLD chemistries are examined in this work (Figure 2), which have a substantial, if not dominant, presence in commercial desalination membranes.^9^ M-phenylenediamine (MPD) and trimesoyl chloride (TMC) form a fully aromatic, cross-linked polyamide (MPD-TMC), which is commonly used for reverse osmosis (RO) membranes (e.g., FilmTec XLE, Dupont). Piperazine (PIP) and TMC form polypiperazine amide (PIP-TMC), which is a semiaromatic, cross-linked polyamide typically used in nanofiltration membranes (e.g., FilmTec NF270, Dupont). These polymers are highly networked due to cross-links formed with each TMC unit due to its three functional groups.

We previously demonstrated that MPD-TMC MLD films with thickness on the order of a few nanometers had similar desalination performance as commercial membranes with overall film thicknesses >100 nm.^8^ This outcome raises several questions: (1) Why did such a thin film have performance metrics similar to those of thicker membranes made from the same material? (2) Do the films have the same structure? (3) Is there a difference in cross-linking? Our work now shows that compared to the literature and commercial IP films, MLD creates polyamides that are denser and fully cross-linked. This work provides insights into the MLD growth mechanism and investigates the roles of surface functional groups and double reactions during MLD.

This study begins with demonstrating MLD growth of MPD-TMC and PIP-TMC films on silicon substrates, followed by a characterization of their composition, cross-linking, bulk morphology, density, and surface morphology. These results reveal the relationship between temperature, cross-linking, and rate of film growth with MLD. The nanoscale morphology of IP and MLD films is compared using grazing-incidence wide-angle X-ray scattering (GIWAXS). Finally, we discuss the similarities and differences between IP and MLD films and how these differences impact their effectiveness or potential as desalination membranes. Altogether, these results provide new and important knowledge for membrane materials science, the development of MLD membranes, and a better understanding of the MLD process.

## 2 Experimental Section

### 2.1 Spatial Molecular Layer Deposition

**Figure 3:**
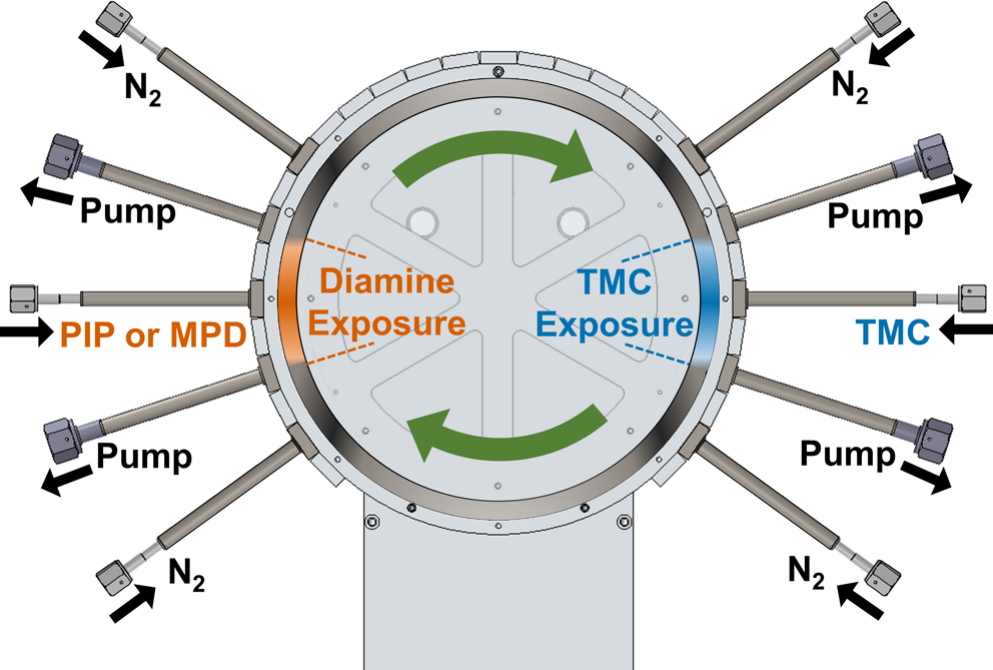
This cross-sectional diagram of the spatial MLD system shows how samples are cycled between two reactant exposure zones. Adapted with permission from ref (8). Copyright 2021 Elsevier.

Polyamide thin films were grown in the custom-built spatial MLD system shown in Figure 3. The MLD process was performed by exposing substrates to two reactive precursors in an isolated, sequential manner. This was facilitated by two precursor exposure zones that were created in the 1 mm gap between two concentric drums. One reaction zone contained TMC (1,3,5-benzenetricarbonyl trichloride) (98%, Sigma-Aldrich), while the other contained either MPD (99%, Sigma-Aldrich) or PIP (99%, Sigma-Aldrich). These regions of exposure were each maintained by five flow modules that were mounted on the outer drum. Precursors flowed into each exposure zone through the center module. The two adjacent modules were connected to a vacuum pump where unused reactants, gas byproducts, and nitrogen were removed from the system. The outer two modules were used to introduce nitrogen gas (4.8 grade, Airgas). Nitrogen acted as an inert gas diffusion barrier for the confinement of precursors within the reaction zones. Substrates were mounted to the inner drum (Figure 3) using Kapton adhesive tape. The substrates moved between exposure zones by rotation of the inner drum. No chemistry occurred in the purge zones, i.e., the regions between the exposure zones that contained no reactants. As configured, one MLD cycle is performed per rotation, exposing the substrate to each precursor once. Details of the spatial MLD system are provided in the Supporting Information, Section A. Further information on the spatial system is provided in refs (8,10−14).

### 2.2 Spectroscopic Ellipsometry

Film thickness was measured by using spectroscopic ellipsometry (M-2000, J. A. Woollam Co., Inc.). Samples grown on reflective silicon and titanium were analyzed at three incident angles: 50, 60, and 70° with the CompleteEase software (J. A. Woollam). The PIP-TMC spectra were fit by using a Tauc–Lorentz model, while Gaussian oscillators were used to fit the MPD-TMC spectra. Both models were developed with multisample analysis.

### 2.3 X-ray Reflectivity

A Jordan Valley/Bede D1 X-ray diffractometer was used for X-ray reflectivity (XRR) measurements of the MLD films on silicon substrates. X-rays from a Cu Kα source were used to take reflectivity curves at the specular condition over a scattering vector range of 0.012–0.277 Å^–1^. An off-spectral background curve was collected for each sample and subtracted from the data. Using the GenX software package, the background-subtracted data were fit to an electron density profile model, which included footprint correction for X-ray beam overspill.^15^

A five-layer model was used, which consisted of the silicon substrate, a native silicon oxide, a bottom interfacial polyamide stratum, a bulk polyamide stratum, and a top polyamide stratum. The native silicon oxide thickness was set to 1.5 nm, and its electron density was fixed at a value corresponding to a mass density of 2.25 g/cm^3^.^15^ Thickness, scattering length density, and interfacial roughness of the three polyamide strata were then fit to the experimental XRR data. The mass density of the films was calculated from the electron density by assuming a composition stoichiometry corresponding to a completely cross-linked film structure.

### 2.4 X-ray Photoelectron Spectroscopy

Compositional analysis was performed with a PHI 5600 X-ray photoelectron spectrophotometry (XPS) unit with a monochromated Al Kα X-ray source. The binding energy was calibrated to 285 eV. Measurements were performed on PIP-TMC (1.2 μm, 120 rpm, 115 °C) and MPD-TMC (101 nm, 20 rpm, 115 °C) MLD films grown on silicon substrates.

### 2.5 Attenuated Total Reflectance Fourier Transform Infrared Spectroscopy

A Cary 630 FTIR spectrometer (Agilent) was used to perform attenuated total reflectance Fourier transform infrared spectroscopy (FTIR) measurements of MLD films on silicon substrates in the range of 650–4000 cm^–1^. Baseline subtraction was performed with OriginPro (OriginLab Corporation). All spectra were scaled for comparison.

### 2.6 Grazing-Incidence Wide-Angle X-ray Scattering

Two-dimensional GIWAXS patterns were measured at the Advanced Photon Source (APS) on beamline 8-ID-E and at the National Synchrotron Light Source II (NSLS-II) at the complex material scattering (CMS) beamline.^16^ Measured samples included MLD films grown on silicon as well as as-received and “rinsed” commercial XLE and NF270 FilmTec thin-film composite (TFC) membranes with the same MPD-TMC and PIP-TMC chemistries, respectively. The rinsed membranes were submerged for 30 min in a 50% (volume) isopropanol/water solution to remove any preservatives and then rinsed with water and air-dried before measurement.^17^ Unless otherwise stated, measurements were performed at APS in air at an X-ray energy of 10.92 keV via a Pilatus 1 M detector with a sample detector distance of 217 mm at incident angles of 0.20° for 4 or 10 s. The as-received MPD-TMC commercial membrane was measured at an incident angle of 0.18° during a different measurement series. While the exact incident angles are reported for completeness, we found that the measurements taken in the range of 0.16–0.20° had negligible differences, as they were sufficiently above the critical angle (∼0.11°) that refraction was not important. Generally, images at two detector positions were combined to remove the horizontal detector gaps using the GIXSGUI toolbox in MATLAB.^18^ The as-received and rinsed NF270 membranes were measured at NSLS-II in air at an X-ray energy of 13.5 keV with a customized Pilatus 1 M detector at a sample–detector distance of 256.99 mm with an incident angle of 0.20° for 10 s.

Python packages pyfai and pygix were used to mask, transform, and integrate the 2D images. These data were corrected for the solid angle, and the intensity was multiplied by sin|chi| to correct for lateral isotropy in the film.^19^ The 1D data were background-subtracted with a fitted linear background and an exponential decay at low *Q*; these data were normalized to the peak maximum between 1.0 and 2.5 Å^–1^ for easy comparison of peak positions and shapes.

### 2.7 Atomic Force Microscopy

An atomic force microscope (AFM) (NX10, Park Systems Corp.) was used in noncontact mode using an OMCL-AC160TS (Olympus Corporation) cantilever with a nominal spring constant of 26 N/m, a resonance of 300 kHz, and a nominal tip radius of 7 nm. Measurements were performed on MLD films deposited on silicon substrates. To measure the root-mean-square (RMS) roughness of the MLD films, two samples of each polyamide chemistry were scanned in randomly chosen areas. The MPD-TMC samples had film thicknesses of 238 and 1836 nm. The PIP-TMC samples had film thicknesses of 260 and 1237 nm. For each sample, measurement was performed two or three times in 1 μm × 1 μm areas with a 256 × 256 pixel resolution. The RMS roughness of an uncoated silicon sample was also measured in two 5 μm × 5 μm areas with a 256 pixel × 256 pixel resolution. Reported RMS roughnesses and errors were calculated from the mean and standard deviation of the scans. The AFM images were processed by using Gwyddion software (version 2.59).

## 3 Results and Discussion

### 3.1 Characterization of Film Growth

**Figure 4:**
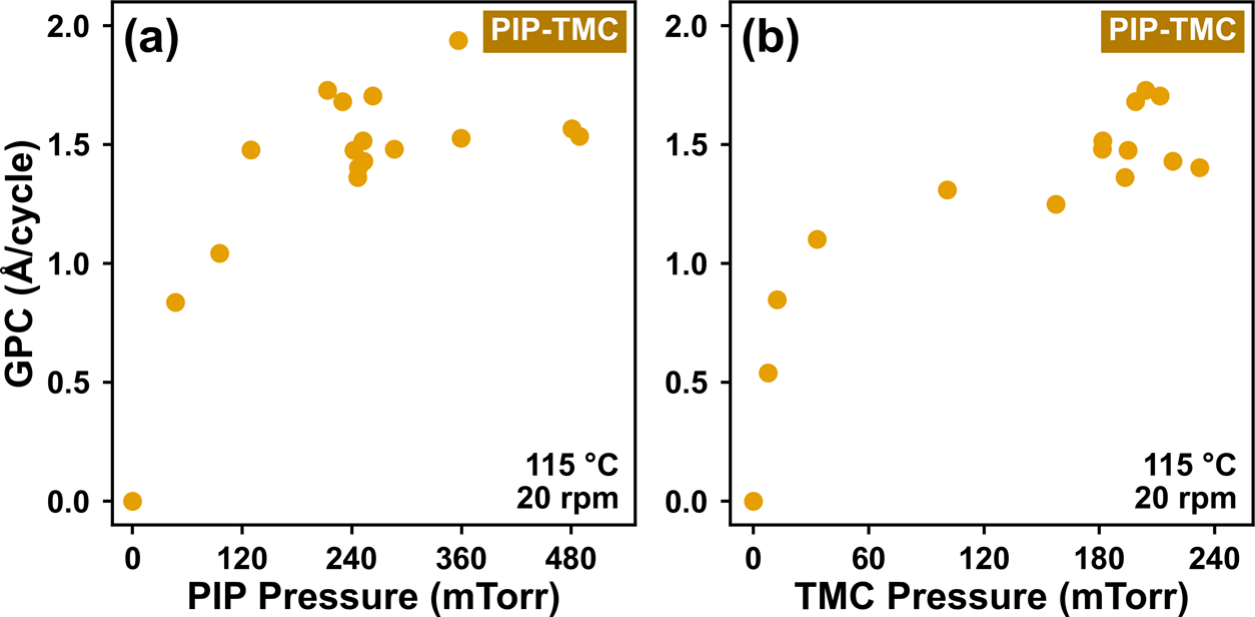
Saturation curves for increasing pressures of (a) PIP and (b) TMC. Growth per cycle (GPC) increased until a growth rate of ∼1.5 Å/cycle, which represents the point at which all substrate functional groups have reacted with the precursor.

MLD film growth of PIP-TMC and MPD-TMC was affected by precursor exposure and the number of cycles. A saturation point exists with every precursor exposure step (Figure 1) in which the substrate surface has experienced a sufficient flux of reactants to react with all substrate functional groups.^20^ Below the saturation point, the growth per cycle (GPC) decreases due to incomplete reactions. Above the saturation point, the GPC is constant. To determine the saturation pressures, MLD film thickness on silicon was measured with ellipsometry for two series of samples (Figure 4). In the first series, the PIP pressure was varied (0–490 mTorr), while the TMC pressure was maintained (206 ± 25 mTorr). In the second series, TMC was varied (0–232 mTorr) and PIP was maintained (250 ± 37 mTorr). In both cases, the GPC increased with pressure until reaching a plateau of ∼1.5 Å/cycle, which indicated that saturation occurred at about 180 and 200 mTorr for TMC and PIP, respectively. Saturation experiments for the MPD-TMC chemistry were previously reported for this system.^14^

A small amount of variance can be seen in Figure 4, which is not uncommon to all-organic MLD; the PIP-TMC data herein are consistent with other MLD chemistries reported in the literature.^21^ This variance could arise from several different sources, including challenging ellipsometry modeling (absorbing films) or inconsistent growth from physisorbed species.^22^ It could also arise from the precursor carryover from one reaction zone to another in small amounts that vary according to the thickness of the polymer film accumulated on the rotating drum over several reactions.

**Figure 5:**
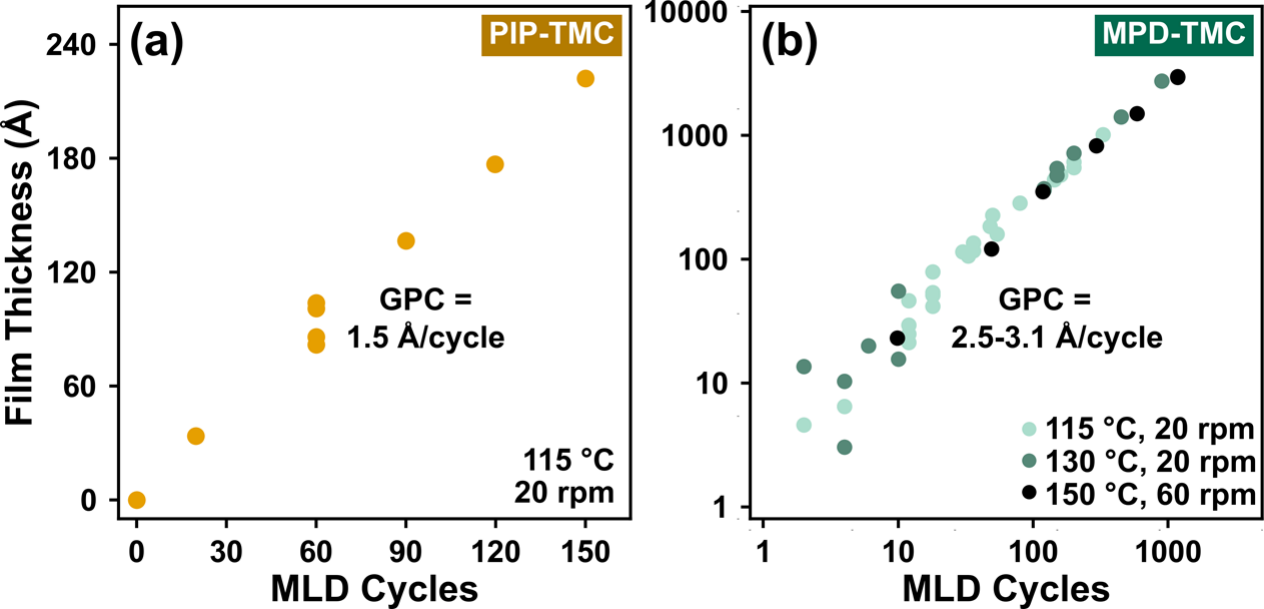
Film thickness scaled linearly with increasing cycles of MLD for (a) PIP-TMC and (b) MPD-TMC polyamides.

To demonstrate the linear growth behavior of MLD, the film thickness was measured with ellipsometry for samples with an increasing number of MLD cycles, as shown in Figure 5. The PIP-TMC chemistry had a GPC of 1.5 Å/cycle on silicon substrates at a rotation speed of 20 rpm and a reaction temperature of 115 °C. The MPD-TMC chemistry was studied at three different conditions. The GPC on titanium substrates at 150 °C and 60 rpm was ∼2.5 Å/cycle. For 130 °C and 20 rpm, GPC on titanium was ∼3.1 Å/cycle. At 115 °C and 20 rpm, the GPC on silicon was ∼2.9 Å/cycle. Higgs et al. reported a growth rate of 4.5 Å/cycle at 115 °C and 20 rpm on titanium in the same system based on a Cauchy ellipsometry model.^14^ The lower values in our study are based on a general oscillator ellipsometry model, which, unlike the Cauchy model, is suitable for analyzing absorbing films.^23^ The axes of Figure 5b are logarithmic to show the entire span of the collected data. The ability to produce many samples with hundreds of MLD cycles is owed to the rapid process rate of the spatial reactor design.

### 3.2 Cross-Linking Analysis

**Figure 6:**
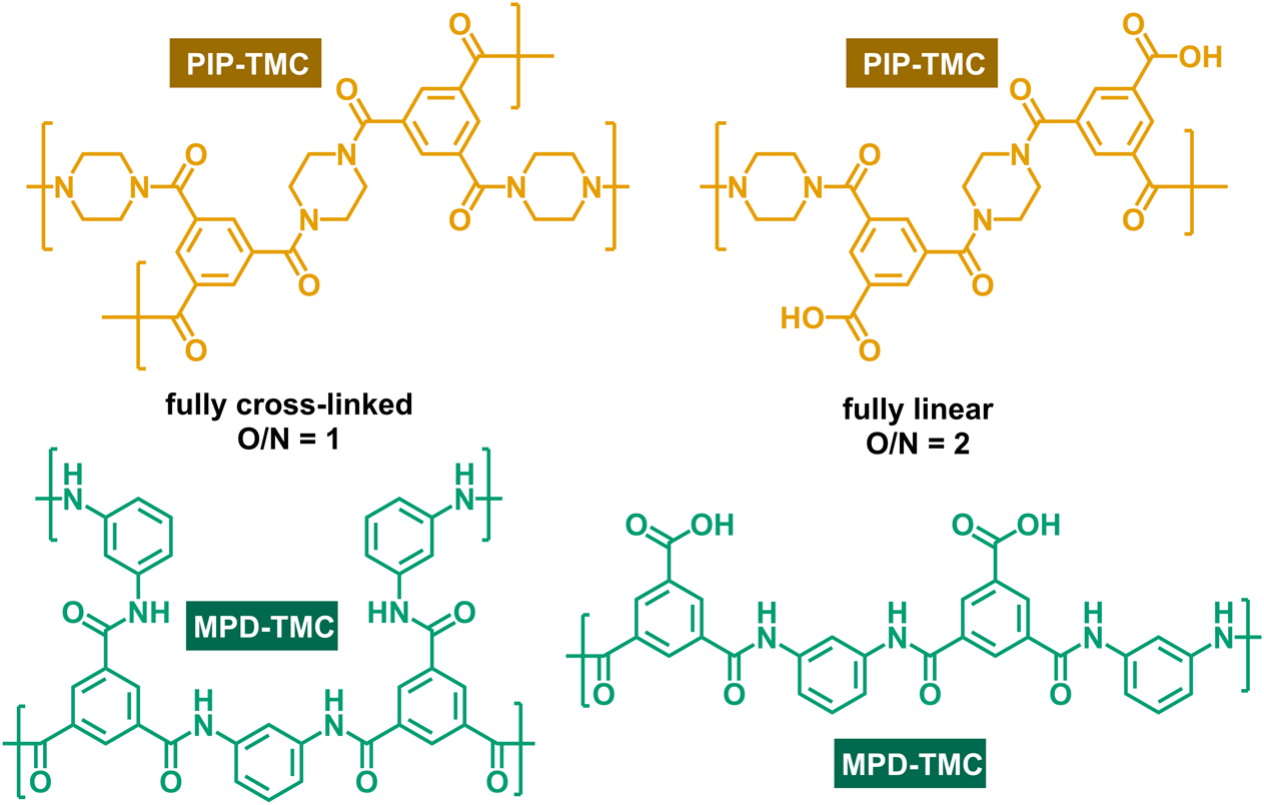
Repeat groups for PIP-TMC and MPD-TMC polyamides. Fully cross-linked structures are the result of reactions between every functional group. Fully linear structures would form in the hypothetical case, where one acyl chloride of every TMC is hydrolyzed.

A high degree of cross-linking is a key attribute for lowering the permeability of solutes and solvents in membrane applications.^24^ The stepwise saturation of MLD reactions provides every substrate amine group with an excess of TMC and every substrate acyl chloride group with an excess of MPD. Therefore, complete cross-linking is expected in the MLD films and can be estimated from the oxygen-to-nitrogen ratio (O/N) of XPS scans, which are shown in Table 1.^25^ The ratios for both of the MLD polyamides were close to unity, which indicated fully cross-linked structures as depicted in Figure 6. IP polyamide films commonly have O/N > 1, indicating a reduced degree of cross-linking due to unreacted acyl chloride groups, which hydrolyze into carboxyl groups.^26^ The boundary case of a fully linear film would result in a ratio of 2, in which all cross-links have been replaced by carboxyl groups (Figure 6). Compositional results from XPS analysis are detailed in Supporting Information, Section B.

**Table 1:** O/N Ratios of the Desalination Polyamides Measured by XPS

	MLD samples	commercial IP (from literature)	fully cross-linked, theoretical	fully linear, theoretical
PIP-TMC	0.99	1.06–1.42^27,28^	1	2
MPD-TMC	1.09	0.96–1.40^27,28^	1	2

**Figure 7:**
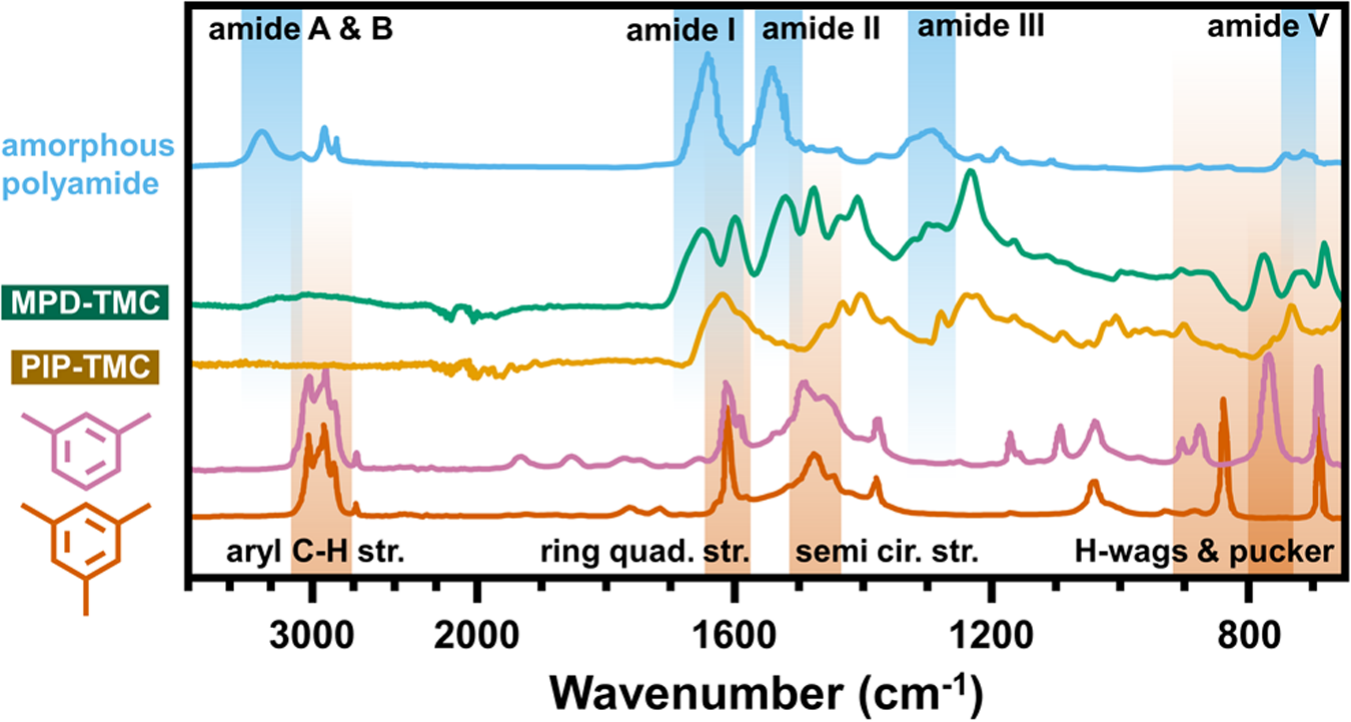
FTIR spectra of MPD-TMC and PIP-TMC polyamide films grown on silicon. FTIR spectra from the literature are shown for comparison, which include amorphous polyamide (hexamethylenediamine-phthalic acid isomers), *m*-xylene, and mesitylene.^29,30^

The use of O/N as an approximation of cross-linking is reasonable for IP, which involves an environment of excess TMC: unreacted amines are highly unlikely, and, as a result, all missing cross-links can be accounted for as carboxyl groups.^25^ To establish a basis for use of the O/N ratio with MLD films, FTIR analysis in Figure 7 confirmed that the MLD films contained no other nitrogen- or oxygen-containing groups besides the moieties of the fully cross-linked structures (Figure 6). Both MPD-TMC and PIP-TMC lacked any discernible carboxyl signal in the 1700–1730 cm^–1^ region. Furthermore, no N–H signals were found in the PIP-TMC film, which would have resulted from the presence of unreacted PIP. Full FTIR analyses including peak assignments are detailed in the Supporting Information, Section C. For Figure 7, mesitylene and m-xylene are shown as comparative models for the aromatic moieties of TMC and MPD, while amorphous polyamide (hexamethylenediamine-phthalic acid isomers) demonstrates the secondary amide moieties.^29,30^

### 3.3 Surface Morphology

AFM analysis showed that MLD films are smooth and conformal in comparison to the rough, ridge-and-valley morphology of IP films. Roughness measurements of MLD films on silicon substrates are summarized in Table 2, and scan images may be found in the Supporting Information, Section D. The films were conformal and smooth with no statistical differences found in the RMS roughness between the silicon substrate and either the MPD-TMC film (*t*-value = 0.96, degrees of freedom = 6, *p*-value >0.05, per Student’s *t* test) or the PIP-TMC film (*t*-value = 0.53, degrees of freedom = 5, *p*-value >0.05). As a result of this conformality, the ultimate roughness and morphology of any membranes fabricated using MLD will depend on the support layer rather than the film deposition technique. Such a feature is compelling for the potential creation of engineered membrane surfaces.

**Table 2:** AFM-Measured Surface Roughnesses of MLD Polyamide Films on Silicon Substrates and Uncoated Silicon

sample	RMS roughness (nm)
silicon (bare substrate)	1.2 ± 0.4
PIP-TMC film	0.9 ± 0.8
MPD-TMC film	1.1 ± 0.6

IP films have morphology consisting of folds, voids, peaks, and valleys, which have been attributed to instabilities in the IP process. Freger and Ramon discuss many instabilities in the IP process, which may lead to such morphological features including thermodynamic, kinetic, hydrodynamic, and elastic instabilities.^31^ The rough, ridge-and-valley morphology is known to be susceptible to colloidal fouling.^5,6^ This finding has led to the development of heavily altered IP processes, which may decrease roughness as well as film thickness, e.g., electrospray, molecular layer-by-layer, and substrate-free IP.^32−34^Table 3 reports the order of magnitude for the RMS roughness of the films produced by each technique. MLD does not suffer from the instabilities of these solvent-based techniques and consistently produces smooth, conformal films. From these results, MLD membranes are expected to have excellent resistance to colloidal fouling compared to other films.

**Table 3:** Order of Magnitude for RMS Roughness for Polyamide Film Surfaces Produced by Various Techniques

polymer	film growth technique	substrate	RMS roughness (nm)	source
MPD-TMC	MLD	silicon	1	this work
	MLD	polymeric membrane	1–10	(8)
	IP, commercial	polymeric membrane	10–1000	(29,34,35)
	electrospray	polymeric membrane	1–100	(37)
	IP, free standing	silicon	0.1–10	(32)
	molecular layer-by-layer	silicon	0.1–1	(33,35)
PIP-TMC	MLD	silicon	1	this work
	IP, commercial	polymeric membrane	1–100	(4,29,37)

### 3.4 Film Density and Surface Characteristics

**Figure 8:**
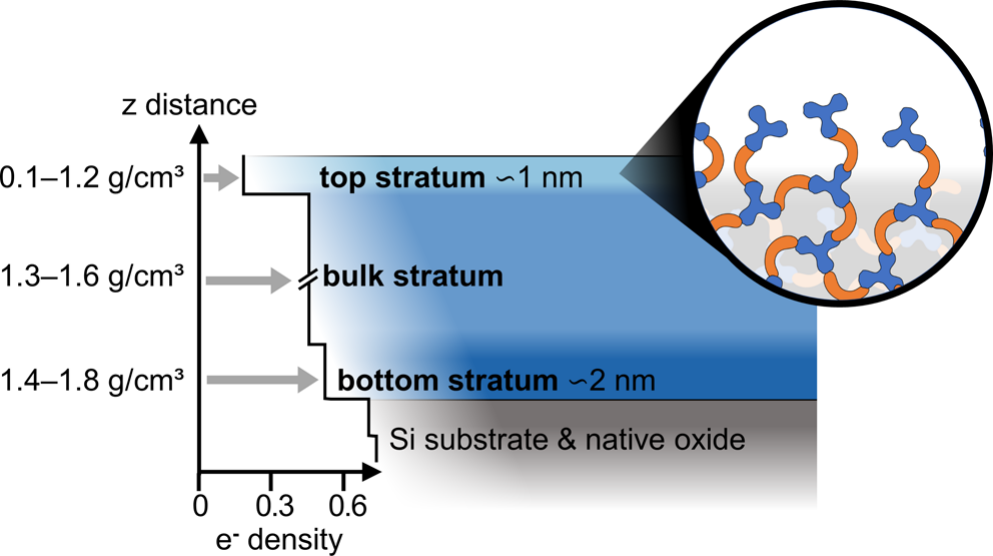
Cross section of a three-strata density structure is illustrated based on the XRR analysis of the MLD polyamide films. The top stratum may be composed of short polymer segments, 1–2 monomers in length.

XRR analysis showed that MLD films had three polymer strata of varying densities: a top stratum, a bulk stratum, and an interfacial stratum (Figure 8). The bulk stratum was the thickest for the MPD-TMC and PIP-TMC MLD films with a density of 1.3–1.6 g/cm^3^. The top ∼1 nm stratum had a low effective mass density (0.1–1.2 g/cm^3^). A bottom ∼2 nm stratum formed near the Si substrate with an increased mass density (1.4–1.8 g/cm^3^), possibly due to an abundance of surface functional groups at the silicon surface (upward of 4.9 hydroxyls/nm^2^) compared to the bulk film.^39^ While the bulk stratum contained a fully cross-linked network, the decreased density of the top stratum may be due to surface roughness or non-cross-linked polymer segments. XRR data, models, and results are detailed in the Supporting Information, Section E.

The MLD polyamide films were denser than their IP counterparts, likely due to the comparatively high degree of cross-linking, as well as a conformal morphology, which suggest a reduction in the volume of voids within the polymer films. The bulk film density of the MPD-TMC MLD films (measured 1.58 ± 0.05 g/cm^3^) was greater than that of IP films, which can range from 1.0 to 1.5 g/cm^3^.^40^ Likewise, PIP-TMC MLD films (measured 1.39 ± 0.08 g/cm^3^) were denser than the IP films (∼1 g/cm^3^).^40^

Analysis of the GPC and the density of the MLD films provided insights into the deposition process. The mass deposition rate was estimated to be 47 ng/cm^2^ per cycle for MPD-TMC and 21 ng/cm^2^ per cycle for PIP-TMC. The MPD-TMC films formed an average of ∼9 amide bonds per nm^3^ or ∼2.7 amide bonds per nm^2^ per cycle, spaced ∼7 Å apart. Likewise, the PIP-TMC films formed ∼9 amide bonds per nm^3^ or ∼1.3 amide bonds per nm^2^ each cycle, spaced ∼9 Å apart. These calculations are detailed in the Supporting Information, Section F. Thus, the PIP-TMC films contain far fewer surface functional groups per unit area than MPD-TMC films.

### 3.5 Molecular Layer Deposition Growth Dependence on Temperature

While increased temperatures are generally known to lead to decreased growth rates, the GPC of the MPD-TMC chemistry had a minimal decrease between 115 and 150 °C (Figure 5b).^41^ To study this effect, MLD growth data for MPD-TMC were compared to previously reported values. The MPD-TMC chemistry had a GPC of 2.9 Å/cycle at 115 °C compared to 2.5 Å/cycle at 150 °C: a ∼0.4% decrease per °C. As shown in Table 4, this value is comparable to cross-linked hybrid organic–inorganic MLD chemistries, which report a decrease of 0.2–1.2%/°C across their maximum reported temperature ranges.^42−46^ In contrast, the GPC for all-organic MLD of non-cross-linked polymers (formed by two bifunctional MLD precursors) decreased at a greater rate: 1.8–5%/°C.^47−52^ To the best of the authors’ knowledge, there are no previously reported temperature studies of the GPC of all-organic cross-linked MLD, and no other all-organic MLD chemistry for which a temperature window of stable growth has been observed.

**Table 4:** MLD Growth Rate Decrease with Increasing Temperature^a^

precursors	material type	structure	decrease in GPC with increasing temperature (%/°C)	source
ED-PMDA	polyimide	non-cross-linked	5.0	(50)
PPD-PMDA	polyimide	non-cross-linked	5.0	(50)
ODA-PMDA	polyimide	non-cross-linked	4.8	(50)
ED-AC	polyamide	non-cross-linked	2.9	(51)
HD-PMDA	polyimide	non-cross-linked	2.5	(50)
PPD-TC	polyamide	non-cross-linked	2.4	(49)
EG-TC	polyester	non-cross-linked	2.2	(52)
HD-PDIC	polyurea	non-cross-linked	2.2	(48)
ED-PDIC	polyurea	non-cross-linked	1.8	(48)
EG-DEZ	zincone	cross-linked	1.2	(46)
EG-TMA	alucone	cross-linked	1.0	(42)
MPD-TMC	polyamide	cross-linked	0.4	this work
GL-TiCl_4_	titanicone	cross-linked	0.3	(44)
EG-TiCl_4_	titanicone	cross-linked	0.3	(44)
HQ-TMA	alucone	cross-linked	0.2	(43)

a Data from figures were extracted using WebPlotDigitizer.^53^ Acronyms are as follows: ED, ethylenediamine; PMDA, pyromellitic dianhydride; PPD, *p*-phenylenediamine; ODA, 4,4′-oxidianiline; HD, 1,6-hexanediamine; AC, adipoyl chloride; EG, ethylene glycol; TC, terephthaloyl chloride; PDIC, 1,4-phenylene diisocyanate; DEZ, diethyl zinc; TMA, trimethylaluminum; GL, glycerol; and HQ, hydroquinone.

There are two exceptions to this trend, which include cross-linked PIP-TMC chemistry and non-cross-linked Nylon-2,6. Unlike the stable growth rate observed with other cross-linked chemistries, PIP-TMC samples, which we grew at 150 °C and 20 rpm, measured decreased growth rates below 0.4 Å/cycle. We attribute this to the decomposition of piperazine.^54^ Myers et al. showed that Nylon-2, 6 MLD films had increasing rather than decreasing growth rates up to a temperature of 67 °C, which was explained by a thermal activation barrier for the reaction.^51^ Even so, the trend of decreasing growth rate was observed above 67 °C.

Why would the decrease in GPC of cross-linked polymers be smaller than that of non-cross-linked polymers? The majority of the surveyed studies have attributed the decrease in GPC with increasing temperature to the precursor-mediated growth mechanism.^45,47−51^ With this mechanism, as temperatures increase, reactions are less likely to occur because the desorption of adsorbed precursors is increasingly favored relative to chemisorption.^47^ Some studies have observed non-self-limiting MLD growth due to precursor absorption.^55,56^ Yet, our survey of data indicates the existence of another mechanism, which generally affects non-cross-linked chemistries to a greater degree than cross-linked.^46,57^ To explain this mechanism, the surface of an MLD polymer may be conceptualized as an entangled mesh of polymer segments. In the case of non-cross-linked MLD, the segments are bonded to the substrate at one end and increase in length with each MLD cycle. For cross-linked MLD, such as MPD-TMC, the segments are short and bonded to the underlying cross-linked bulk film. With each MLD cycle, the surface segments of the cross-linked polymer are incorporated into the bulk, cross-linked stratum, and new segments are formed. To simplify the conceptualization, we ignore the effects of precursor absorption.^22^

**Figure 9:**
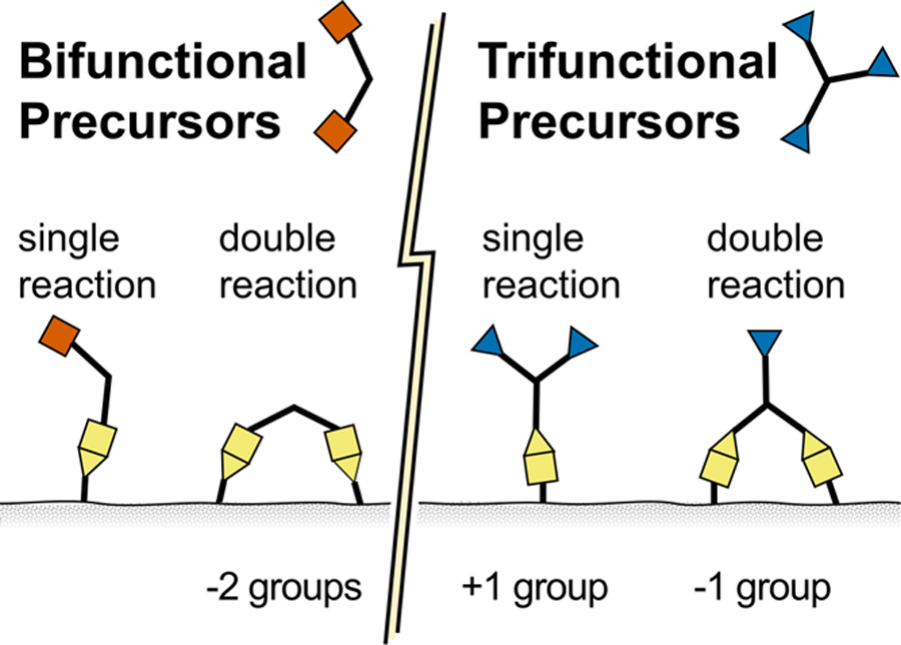
During MLD, precursors may react with functional groups of the substrate once or twice. Single reactions of bifunctional precursors extend the substrate functional groups, whereas the single reaction of a trifunctional precursor adds a functional group. Double reactions, however, reduce the number of substrate functional groups for the two types of precursors.

The flexibility of the polymer segments enables a single precursor molecule to react with two substrate functional groups during MLD.^20,22^ This occurrence, known as a double reaction, leads to the reduction of a substrate functional group for subsequent precursor exposures, as illustrated in Figure 9. Since the growth rate is governed by the number of available substrate functional groups during each cycle, double reactions lead to a reduced growth rate.^58^ Conversely, functional groups may be added to the substrate via a single reaction with a trifunctional precursor such as TMC. Thus, for cross-linked MLD, a balanced number of double reactions and single reactions for each cycle maintains the number of substrate functional groups. However, non-cross-linked MLD chemistries utilize only bifunctional precursors, and therefore, functional groups cannot be added to the substrate, only lost.

**Figure 10:**
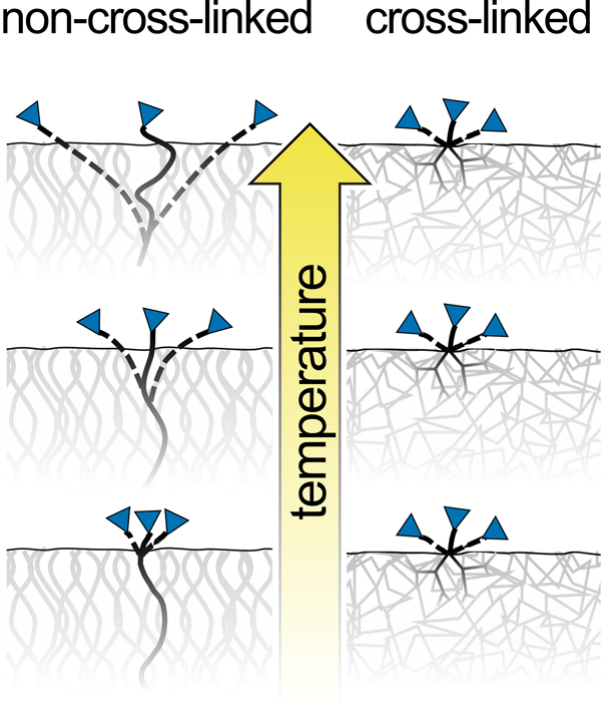
Illustration of the effect of the temperature on the range of motion of substrate functional groups. For non-cross-linked polymer films, the range of motion likely increases with temperature due to increased segmental motion. However, the range of motion is likely constrained by the network structure in cross-linked polymers.

Since double reactions eliminate substrate functional groups, the average distance between the functional groups is a consequence of their range of motion. In other words, if one substrate functional group is positioned within the range of motion of another, a double reaction is likely. Furthermore, segmental motion increases with temperature, which explains the decreasing GPC of non-cross-linked MLD: as the range of motion increases with temperature, the spacing between substrate functional groups increases, enabling a double reaction between the precursor and two neighboring functional groups, as illustrated in Figure 10.^58,59^ Yet, even with increased motion, the ultimate range of segmental motion is limited by the segment length. Therefore, with short polymer segments, cross-linked MLD films may maintain a close spacing of substrate functional groups at all temperatures without the risk of double reactions, resulting in stable GPC of cross-linked MLD.

This conclusion could be further supported if the range of motion of a polymer segment was found to be equal to the average distance between substrate functional groups. To estimate the range of motion, the ∼1 nm extended length of a PIP/TMC or MPD/TMC monomer pair is used, estimated from mean bond lengths and bond angles.^60−62^ This length represents the maximum distance between cross-links and thus the maximum range of an unbound surface segment comprised of up to two monomers. From our analysis of mass densities (Section 3.4), the average distance between amide bonds for PIP-TMC and MPD-TMC was ∼9 and ∼7 Å, respectively. Indeed, the values of the range and spacing are quite close. The ∼1 nm extended length also appears to correlate with the thickness of the loose top stratum modeled by XRR. We speculate that the range of motion of the diamine/TMC pair is responsible for both the top stratum thickness and the spread of the surface functional groups.

**Figure 11:**
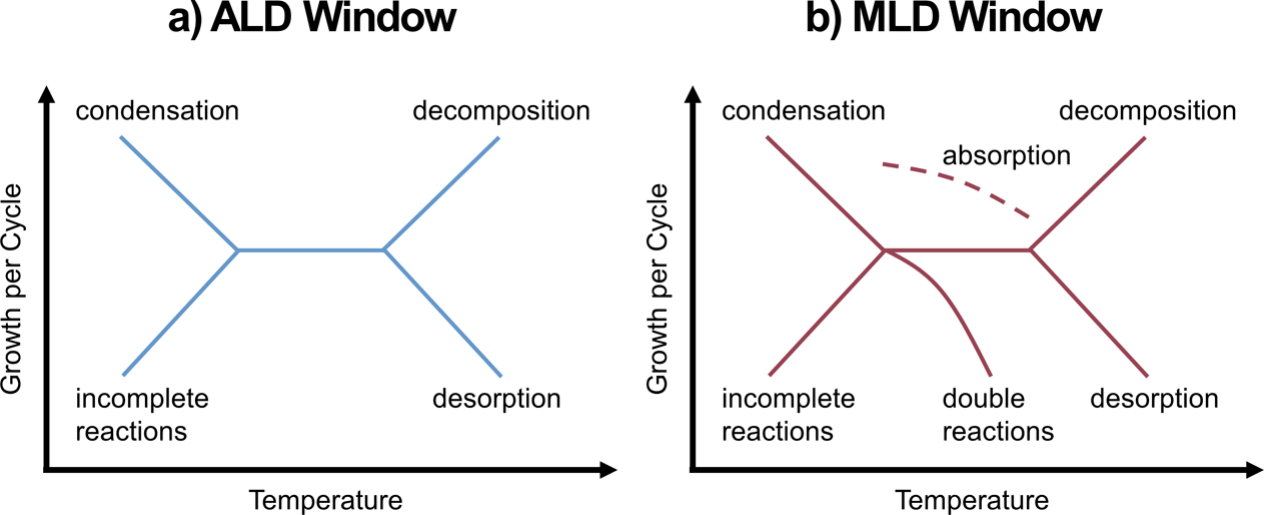
Schematic of the (a) ALD and (b) MLD windows representing the temperature ranges of near-constant growth rates as well as sources of deviation from this growth rate. (a) At the bottom left, decreased growth rates at low temperatures may be attributable to incomplete reactions resulting from slow reaction kinetics. Above this, high growth rates at low temperatures may indicate that precursor condensation has resulted in insufficient purging. At the bottom right, a decreased growth rate at high temperatures may be the result of precursor desorption due to the precursor-mediated growth mechanism. Finally, a high growth rate at high temperatures may indicate that the precursors are decomposing, resulting in uncontrolled growth.^63^ (b) In addition to the effects seen with ALD, MLD growth rate is hindered at increased temperatures due to double reactions. An MLD process may also experience excessive, uncontrolled growth due to the delayed release of precursors, which have absorbed into the MLD film.^55^ Adapted with permission from ref (63). Copyright 2010 American Chemical Society.

With these insights, Figure 11 is proposed as a conceptual plot of the “MLD window” to join the well-known “ALD window”.^63^ ALD and MLD are conceptualized as controlled techniques with a constant growth rate for any given chemistry. By this reasoning, if you were to plot the growth per cycle against the temperature, the data would have a slope of zero. For ALD, this behavior is generally observed but only within a specific temperature region, known as the ALD window. At temperatures outside of this window, the controlled, self-limiting behavior is compromised and the apparent growth rate increases or decreases. The resulting profile of the growth data typically follows one of the paths (lines) shown in Figure 11. Each chemistry has a different temperature window of constant growth and different responses to temperatures outside that range. Figure 11 summarizes the potential temperature-dependent phenomena that cause any given ALD or MLD chemistry to deviate from their constant, controlled growth rate. For ALD (Figure 11a), deviation of the growth can be attributed to condensation or decomposition of reactants, incomplete surface reactions, or premature desorption of precursors (related to the precursor-mediated growth mechanism).^63^ For MLD (Figure 11b), we add two more explanations for deviation from the expected growth rate: (1) double reactions, which progressively decrease the growth rate at higher temperatures, as discussed above, and (2) extra growth from precursors, which are absorbed into the growing MLD film rather than removed during purging.^55,56^

### 3.6 Molecular Packing

**Figure 12:**
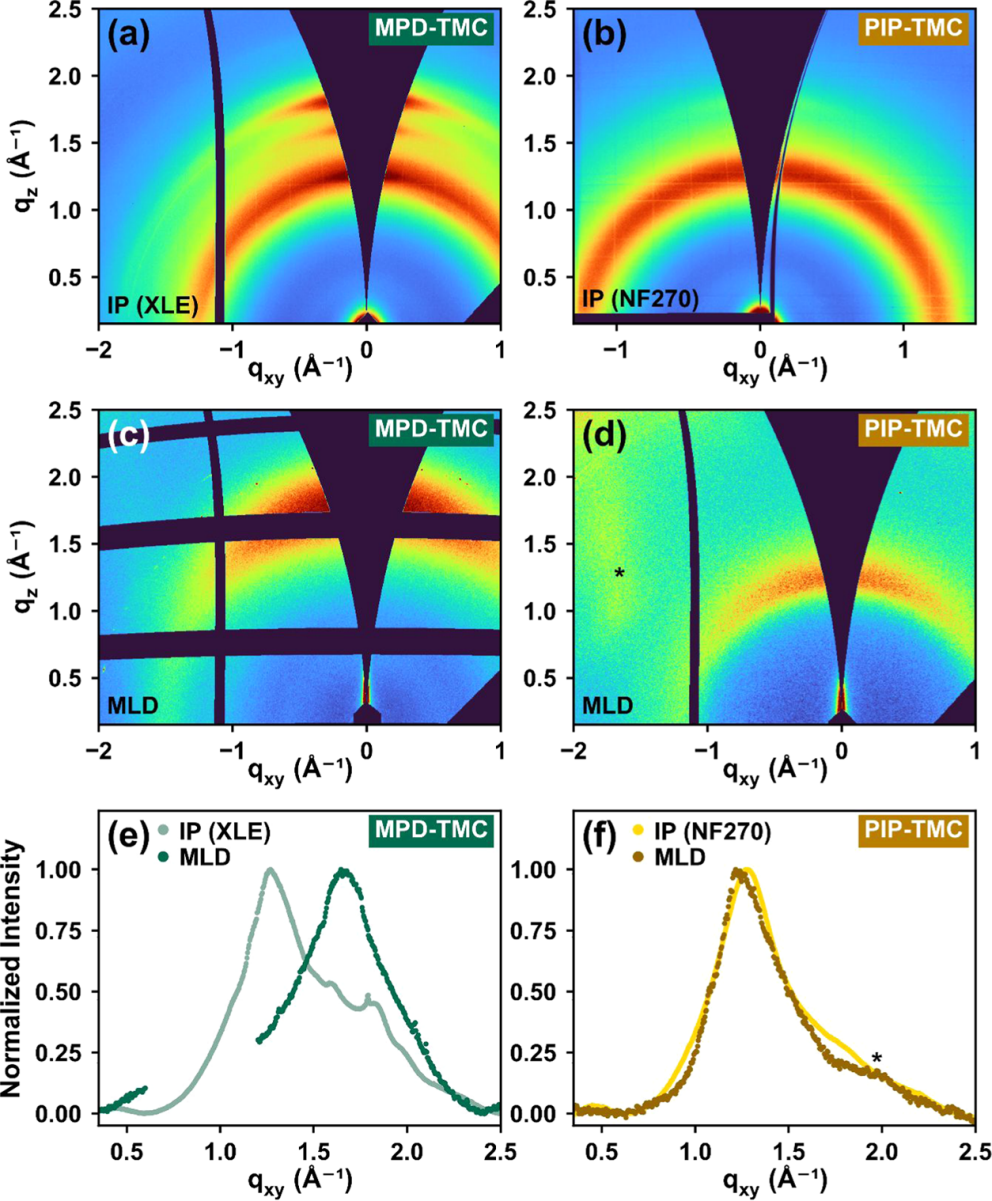
*q_xy_* vs *q_z_* scattering plots for (a) as-received commercial IP MPD-TMC (FilmTec XLE), (b) as-received commercial IP PIP-TMC (FilmTec NF270), (c) MLD MPD-TMC (180 nm, 115 °C), and (d) MLD PIP-TMC (64 nm, 115 °C) films. 1D radial integrations of GIWAXS measurements for (e) MPD-TMC and (f) PIP-TMC chemistries, respectively. * is used in panels (d, f) to highlight a scattering artifact. Note that a different scattering range is used in panel (b) due to measurements being performed at another beamline.

The effect of the synthesis method on molecular packing was investigated using GIWAXS by comparing commercial IP membranes to MLD films with the same chemistry. Our GIWAXS results revealed that MLD films grown on silicon substrates produced structures that were largely consistent with commercial IP films, as shown in Figure 12. The scattering patterns shown in Figure 12e,f demonstrate that MPD-TMC and PIP-TMC film chemistries produced by commercial IP processes and MLD possessed common broad scattering features corresponding to *d*-spacings in the range of 3–5 Å. Scattering images and cake slices for all samples can be found in Supporting Information, Section G.

#### 3.6.1 MPD-TMC

Our GIWAXS measurements of both commercial IP and MLD MPD-TMC films had scattering features around *q* = ∼1.7 Å^–1^, which corresponds to molecular spacings of ∼3.8 Å. However, the commercial IP MPD-TMC film (Figure 12e) showed a distinct peak centered at *q* = ∼1.3 Å^–1^ (*d* = ∼4.9 Å), which was not present in the films prepared by MLD. Fu et al. also observed a similar feature at *q* = ∼1.3 Å^–1^ (*d* = ∼4.9 Å) in their GIWAXS scattering after a citric acid post-treatment of their IP films (presumably to replicate undefined industrial processes).^65^ Thus, this peak may have been the result of postprocessing as opposed to intrinsic differences caused by the synthesis method.

To characterize the relative orientation of these scattering features with respect to the substrate, the peak intensity was analyzed as a function of the polar angle (chi) after applying a sin|chi| intensity correction.^19^ Cake slices taken at 10° increments in Figure S7 revealed that the peak at ∼1.3 Å^–1^ in the commercial membrane, proposed to result from postprocessing, corresponded to features with scattering planes preferentially oriented perpendicular to the substrate. Meanwhile, the higher *q* scattering features, common to all films, were generally more isotropic, consistent with previous findings.^65^

The nanoscale morphology of films with MPD-TMC chemistry has been previously studied using GIWAXS and electron tomography.^4,65,66^ From GIWAXS studies of untreated MPD-TMC films made by the molecular layer-by-layer technique, Fu et al. identified that two peaks (corresponding to *d*-spacings of ∼3.5 and ∼4.0 Å) contained within the broad feature. They assigned these peaks to parallel stacking of aromatic rings. Using electron tomography, Culp et al. revealed nanoscale inhomogeneity of density (regions of high and low densities) and free volume in commercially produced MPD-TMC IP membranes.^4,66^ Insights into the origin of these density inhomogeneities may be provided by other studies of polyamide films. Muscatello et al. performed a coarse grained study of the interfacial polymerization using MPD and TMC monomers, and reported that oligomer clusters polymerize/aggregate until forming a continuous cluster.^67^ They proposed regions with low degrees of polymerization between regions with high degrees of polymerization, consistent with experimental findings.^4^ This led to significant variations in local density, perhaps providing easier diffusion between clusters.^67,68^ Here, it is assumed that molecular ordering is present only in the high-density regions; therefore, our GIWAXS measurements likely did not probe low-density regions.

Comparisons to the literature on the spacings of hydrogen bonds (2.5–3.3 Å), aromatic nylons (1.9–3.0 Å), and the single crystal of reagent MPD (π–HN bonds 2.5–2.8 Å) and π–π stacking may provide some valuable insights into the molecular packing in these membranes.^69−71^ However, the constrained nature of these cross-linked polymers means that computational simulations are likely required to build an understanding of the larger molecular packing spacings (∼3.8 and ∼4.9 Å) in these materials.

#### 3.6.2 PIP-TMC

The PIP-TMC films exhibited a broad scattering peak at *q* = ∼1.3 Å^–1^, corresponding to molecular packing distances of ∼5.0 Å (Figure 12f). This feature was common in all PIP-TMC films, with planes oriented approximately perpendicular to the substrate. Cake slices of the GIWAXS detector images collected for commercial IP and MLD PIP-TMC films are shown in Supporting Information, Section G. The similarity between the scattering profiles of the commercial IP and MLD for the PIP-TMC is interesting, given that these are vastly different synthesis methods.

The average *d*-spacing of the PIP-TMC commercial IP and MLD films measured here was slightly larger than that reported by Singh et al. (∼5.2 Å), who used X-ray scattering to investigate the porosity and structure of fully hydrated PIP-TMC lab-made IP films.^72^ This discrepancy could be a consequence of hydration. We hypothesize that the larger *d*-spacings of the PIP-TMC (∼5.0 Å) compared to those of MPD-TMC (∼3.8 Å) were due to less favorable packing, whereby the flat, planar structure of the MPD moiety is replaced by boat and chair conformations of the PIP moiety.

### 3.7 Insights into Desalination Performance

An understanding of the material structure of the semipermeable membranes utilized in reverse osmosis and nanofiltration provides insight into their function. We can explain performance differences between IP and MLD films by comparing material properties as summarized in Table 5. We previously showed that an MPD-TMC MLD film with a thickness of only a few nanometers, which was supported by a nanofiltration membrane, had similar selectivity and water permeance to commercial IP films with overall thicknesses of >100 nm.^8^ The high density and high degree of cross-linking of the MLD films were likely responsible for excellent selectivity across such a thin selective layer. In comparison to the dense, conformal MLD films, MPD-TMC IP films are known to have interconnected surface areas with intrinsic polyamide wall thickness as low as 20 nm.^73^ Thus, where the IP films are lacking in nanoscale consistency, the complex morphology appears to compensate with respect to increased active area and interconnectivity, which could also provide a favorable “gutter effect”.^74^ These properties also help explain why the thinner MLD film did not lead to increased flux compared with the thick IP films. Optimization of water transport may be possible by tuning film density and cross-linking through alternative MLD chemistries.

**Table 5:** Comparison of Material Properties of MLD and IP Polyamide Films

	MPD-TMC		PIP-TMC	
	MLD	IP	MLD	IP
overall thickness (nm)	>1	100–300^75^	>1	15–40^75^
density (g/cm^3^)	1.5–1.6	1.0–1.5^40^	1.3–1.4	∼1^40^
O/N ratio	1.09	0.96–1.40^27,28^	0.99	1.06–1.42^27,28^
cross-link density (amides/nm^3^)	9		9	
RMS roughness (nm)	∼1	10–100^28,35,36^	∼1	1–10^8,28,38^
morphology	conformal	folds and voids, peaks and valleys	conformal	folds and voids, peaks and valleys
size of ordered domains	∼3.8 Å	∼3.8 and ∼4.9 Å	∼5.0 Å	∼5.0 Å
	planes weakly oriented perpendicular to the substrate	some planes oriented perpendicular to the substrate	planes perpendicular to the substrate	planes perpendicular to the substrate

While commercial NF270 membranes were suitable supports for MLD membranes, the use of more permeable and temperature-stable supports will likely improve performance and facilitate process scale-up.^8^ Such a support would also enable the creation of nanofiltration membranes with PIP-TMC MLD. We expect such PIP-TMC membranes to have a higher rejection of multivalent and monovalent ions but a lower flux compared to IP membranes based on the high degree of cross-linking and high density of the PIP-TMC MLD films. The FTIR and XPS analyses showed that unlike IP films, the PIP-TMC MLD films lacked the presence of internal N–H or COOH groups, which play a significant role in hydrogen bonding within the polymer. Through molecular dynamic simulations, Zhang et al. have shown that such hydrophilic groups inhibit water transport in polyamide membranes.^76^ These results predict that the hydrophobic structure of PIP-TMC MLD films could optimize flux.

As a replacement for IP, MLD membranes may provide significant benefits beyond performance improvements. The spatial MLD reactor utilized in this study was able to process samples at rates of up to 120 m/min with a maximum width of 30 cm. Industrial MLD tools are currently available that can perform deposition on wider samples in roll-to-roll configurations and at atmospheric pressures, allowing rapid, continuous processing of precisely controlled thin films.

While these commercial-scale tools exist, a scalable technique for creating and fabricating MLD TFC membranes has not yet been developed, owing to the propensity to deposit on pore walls, rather than across the pore openings.^13,77^ Another major challenge to commercialization includes the low vapor pressure of the precursors that necessitates a near-isothermal, high-temperature (>100 °C), vacuum environment, which limits the substrate selection and increases manufacturing cost and complexity. For these reasons, MLD membranes may become more marketable to niche, high-value separation applications before widespread use in desalination plants. Regardless, the scale-up of MLD membranes on polymeric substrates is attainable and the prospect of overcoming current challenges is promising.

## 4 Conclusions

In this study, we synthesized and characterized MPD-TMC and PIP-TMC films grown by MLD and compared their characteristics to those of commercial IP desalination films. The top ∼1 nm of MLD films was less dense than the bulk of the film and was likely comprised of loose polymer segments up to two monomers long, which were anchored to the cross-linked bulk layer. These short segments, along with additional functional groups provided by the trifunctional TMC precursor, gave the MPD-TMC chemistry a temperature window of stable, self-limited growth. This is a contrast to non-cross-linked MLD chemistries, which decrease in GPC with increasing temperature due to increasing segmental motion, which may lead to a greater extent of double reactions.

The PIP-TMC MLD films showed a remarkable degree of similarity to the IP film in terms of the molecular structure and orientation of the ordered domains. Both synthesis techniques produced films with ordered domains consisting of planes spaced ∼5.0 Å perpendicular to the substrate. However, PIP-TMC MLD films were more cross-linked and denser than their counterpart IP films. These attributes, in addition to the lack of internal hydrophilic groups, predict PIP-TMC MLD films to have improved permeance/selectivity compared with IP membranes.

Compared to IP, MPD-TMC MLD films were conformal in terms of film thickness and smooth in terms of morphology. They also measured an larger mass density, likely due to a reduction in the volume of voids within the film. These observations explain the high rejection/low permeance performance observed in exceptionally thin MLD membranes. Unlike the MLD films, we observed a scattering plane spaced ∼4.9 Å in the commercial IP film, which we hypothesize to be a result of postprocessing steps. Both MLD and IP films had ordered domains with scattering planes spaced ∼3.8 Å, oriented generally perpendicular to the substrate. This ∼3.8 Å spacing of planes is less than PIP-TMC planes (∼5.0 Å), likely due to steric differences between the MPD and PIP moieties: the MPD moiety is generally flat and planar, while the PIP moiety contains boat and chair conformations.

The insights into MLD growth rates and surface mechanics provide an improved understanding of the MLD processes and enable further development of MLD applications, especially membrane separations. This study also provides a deeper understanding of the molecular structure of MLD and IP films and contributes to understanding the synthesis and performance of reverse osmosis and nanofiltration membranes.

## Supporting Information Available

Design and operation parameters of the spatial MLD system; XPS compositional results of MLD films; FTIR peak assignments for MLD films; AFM images of silicon and MLD films; XRR modeling results for thickness and density; calculations for MLD mass deposition rate, amide bond density and spacing; and X-ray scattering plots of MLD and IP films (PDF)
