# Supplementary material for: Building Semipermeable Films One Monomer at a Time: Structural Advantages via Molecular Layer Deposition vs Interfacial Polymerization
Source: Chem Mater. 2024 Jan 18;36(3):1362–74. doi: 10.1021/acs.chemmater.3c02519 (PMC10870709; doi:10.1021/acs.chemmater.3c02519)
Supplement: Supplementary file 1 — cm3c02519_si_001.pdf [file cm3c02519_si_001.pdf]

Supporting Information

Building Semipermeable Films One Monomer at a Time: Structural Advantages via Molecular Layer Deposition vs Interfacial Polymerization

Brian C. Welch [a,b], Emma N. Antonio [b], Thomas P. Chaney [b], Olivia M. McIntee [b], Joseph Strzalka [c], Victor M. Bright [b], Alan R. Greenberg [b], Tamar Segal-Peretz [a], Michael Toney [b], and Steven M. George [b]*

[a] Israel Institute of Technology, Haifa 3200003, Israel
[b] University of Colorado, Boulder, Colorado 80309, USA
[c] Argonne National Laboratory, Lemont, Illinois 60439, USA

*email: bw@campus.technion.ac.il

A. Spatial Molecular Layer Deposition

Two substrates were used to analyze film growth and material characteristics. MPD-TMC MLD films grown at 130°C and 150°C were deposited on silicon substrates or metalized polyethylene naphthalate (PEN) (ROWO Coating). The metal coating was sputtered titanium (∽80 µm) which served as a reflective surface for ellipsometry. The narrow space between the inner and outer drums permitted use of silicon coupons with a thickness <400 µm and a width of ∽5 mm.

Precursors were stored in custom stainless-steel cylinders. Precursor flow was controlled by use of needle valves. Nitrogen flow was regulated with a mass flow controller (Alicat, MC-1SLPM-D) whose total flow was divided between the four nitrogen modules. The pneumatic isolation valves (Fujikin) contained elastomer seals made from perfluoroalkoxy alkane (PFA) to prevent degradation in the presence of amines and acyl chlorides.

The pressure of the spatial reactor was monitored with capacitance monometers (121A Baratron®, MKS). One monomer was placed at each dosing line and a third measured the pressure of the drum. Precursor pressures are reported as the mean difference between the dose pressure and base pressure for the respective dosing line during reaction.

The inner drum was rotated about a shaft which was coupled to a stepper motor (MDrive23Plus, Schneider Electric). Speeds of up to 120 rpm were achieved. For a constant rotation speed, ω (rpm), residence time in each exposure zone (36°) was equal to 6/ω (s). The residence time in each purge zone (144°) was equal to 24/ω (s).

The entire MLD system (excluding the pumps and exhaust) was placed inside a custom convection oven to ensure near isothermal conditions for the chamber, tubing, fittings, valves, and storage cylinders. Temperature was maintained with proportional-integral (PI) control. To ensure steady state temperatures, heating and cooling times of at least 10, 12 and 24 h were performed before running MLD reactions at 115, 130 and 150°C, respectively.

B. X-Ray Photoelectron Spectroscopy Analysis

The XPS measurements of PIP-TMC (1.2 µm, 120 rpm, 115°C) and MPD-TMC (101 nm, 20 rpm, 115°C) samples provided elemental compositions which are shown in Figure S1 and summarized in Table S1. Carbon content levels above the stoichiometric values were attributed to adventitious species. A low presence of fluorine was attributed to contamination from the fluorocarbon vacuum grease used to lubricate the shaft seals of the reactor. The theoretical stoichiometric composition was calculated from the fully crosslinked polymer repeat units (Figure 6).

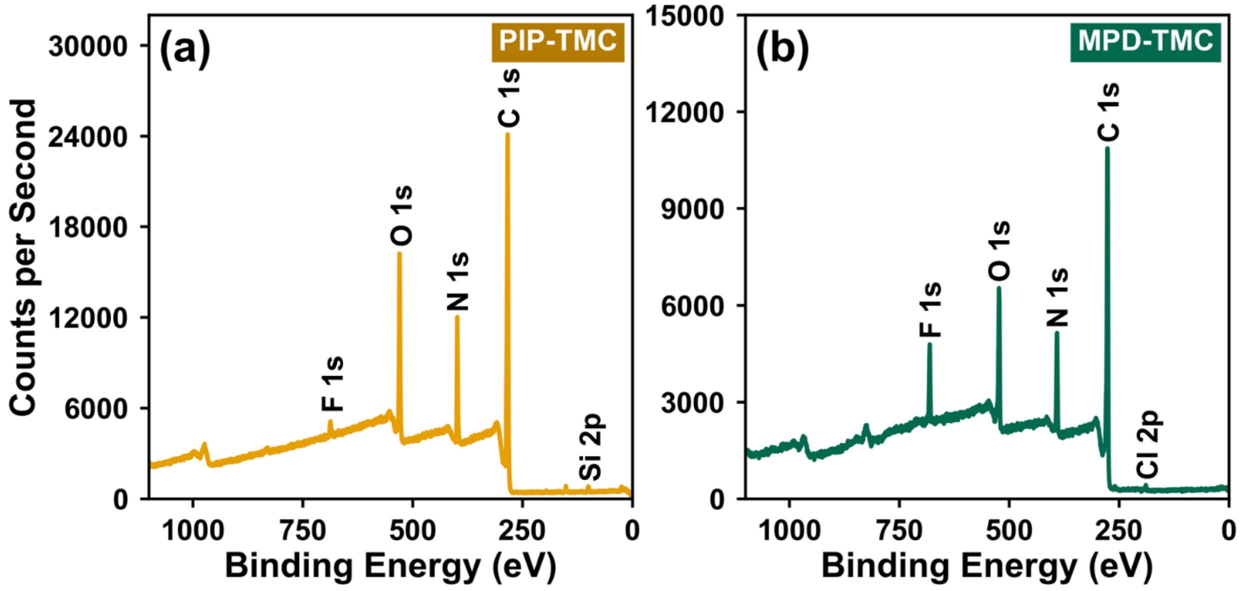

Figure S1. *XPS spectra of (a) PIP-TMC and (b) MPD-TMC MLD films.*

Table S1. *Composition of MLD Polyamides by XPS Analysis*

	**PIP-TMC**		**MPD-TMC**	
	**Stoichiometric**	**Measured**	**Stoichiometric**	**Measured**
C	71.4 %	74.7 %	75.0 %	75.3 %
O	14.3 %	11.7 %	12.5 %	10.8 %
N	14.3 %	11.8 %	12.5 %	9.9 %
F	-	0.5 %	-	3.4 %
Si	-	1.3 %	-	0.0%
Cl	-	0.0%	-	0.6 %

C. Infrared Spectroscopy Analysis

The following assignments were given to the PIP-TMC and MPD-TMC spectra shown in Figure 7. Amide I bands were assigned to MPD-TMC at 1650 cm^{-1} and PIP-TMC at 1620 cm^{-1}. The amide II band was assigned to MPD-TMC at 1517 cm^{-1}. Both polyamides showed amide III bands at 1278 cm^{-1} (PIP-TMC) and 1290 cm^{-1} (MPD-TMC). The shoulder of the MPD-TMC spectra at 715 cm^{-1} was assigned to the amide V mode. A weak, broad signal around 3200 cm^{-1} was ascribed to amide A & B stretching for MPD-TMC. The amide II, V, A, and B bands did not appear with PIP-TMC due to the lack of N-H groups.

The aromatic components of the polyamide led to several features in the FTIR spectra. A broad, weak peak around 3000 cm^{-1} (MPD-TMC) was assigned to aryl C-H stretches. Ring quadrant stretching could be seen in the MPD-TMC polyamide at 1598 cm^{-1}. For the PIP-TMC polyamide, the signal overlaps with the amide I band around 1595 cm^{-1}. Ring semi-circle stretch is a component of MPD, but does not appear in the TMC aromatic motif.[1] As such, a corresponding absorbance feature was found in MPD-TMC (1476 cm^{-1}), but does not appear in PIP-TMC. For the MPD-TMC polyamide, the ring pucker (682 cm^{-1}), and adjacent (775 cm^{-1}) and lone (861 cm^{-1}) C-H wags were assigned.[2] The expected ring pucker and lone C-H wags were less discernable for the PIP-TMC polyamide and were not given assignment.

D. Atomic Force Microscopy Analysis

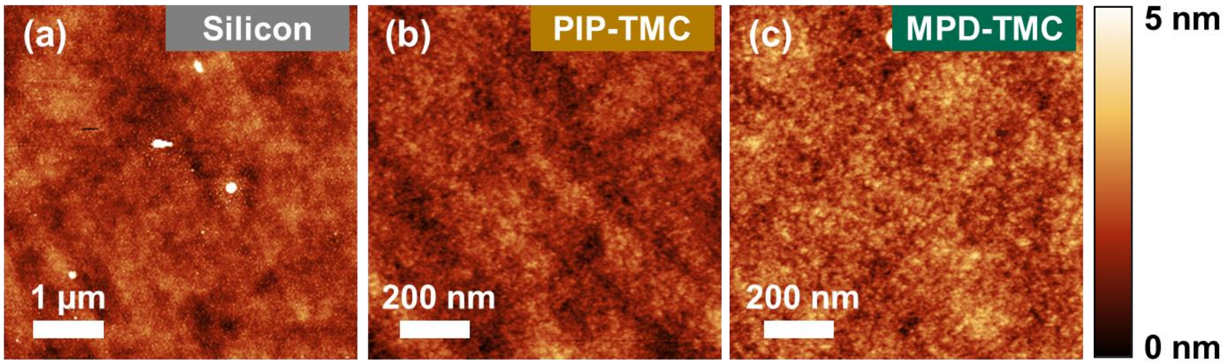

Figure S2. *Representative AFM scans of (a) unprocessed silicon, (b) a 260 nm thick PIP-TMC film and (c) a 238 nm thick MPD-TMC film.*

E. X-Ray Reflectivity Analysis

Table S2. *A Summary of XRR Results for MLD Polyamide Films*

chemistry	MPD-TMC	MPD-TMC	MPD-TMC	PIP-TMC	PIP-TMC	PIP-TMC
temperature	115°C	115°C	130°C	115°C	115°C	115°C
rotation rate	1 rpm	20 rpm	120 rpm	120 rpm	20 rpm	120 rpm
bottom thickness	32 Å	10 Å	13 Å	16 Å	13 Å	13 Å
bulk thickness	162 Å	178 Å	210 Å	143 Å	106 Å	652 Å
top thickness	5 Å	5 Å	5 Å	12 Å	22 Å	34 Å
bottom density	1.7 g/cm³	1.6 g/cm³	1.6 g/cm³	1.6 g/cm³	1.6 g/cm³	1.4 g/cm³
bulk density	1.6 g/cm³	1.5 g/cm³	1.5 g/cm³	1.4 g/cm³	1.4 g/cm³	1.3 g/cm³
top density	0.6 g/cm³	1.2 g/cm³	1.1 g/cm³	0.6 g/cm³	0.1 g/cm³	1.2 g/cm³
bottom roughness	5 Å	4 Å	4 Å	3 Å	5 Å	4 Å
bulk roughness	7 Å	17 Å	17 Å	3 Å	8 Å	8 Å
top roughness	2 Å	5 Å	3 Å	7 Å	3 Å	6 Å

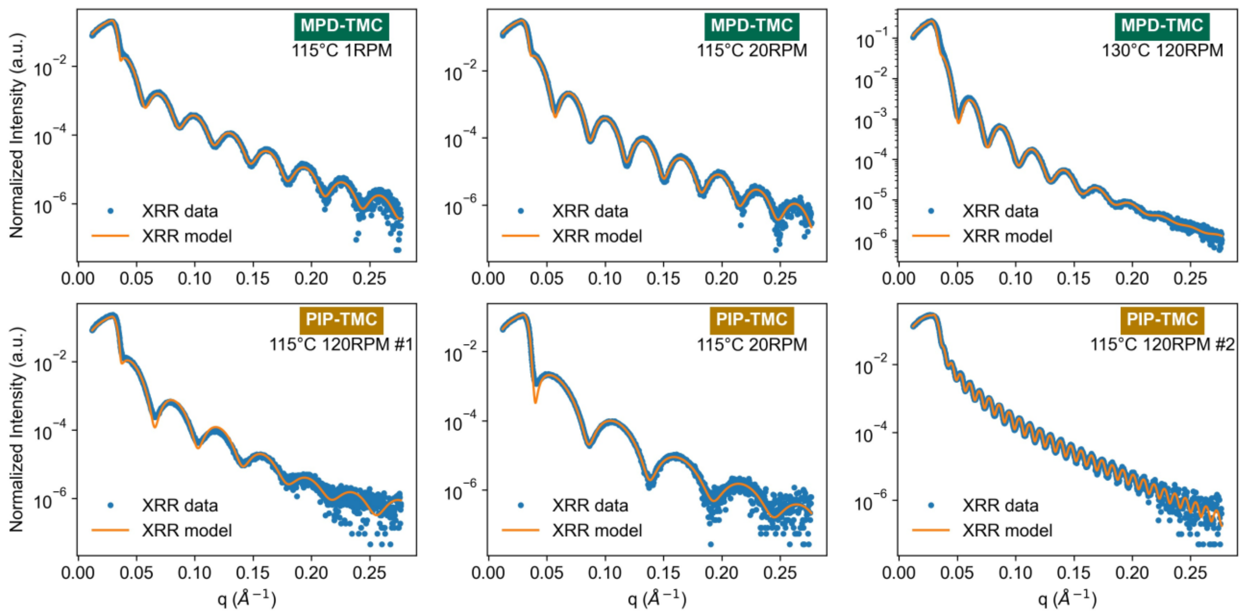

Figure S3. *XRR data and respective fits for each sample.*

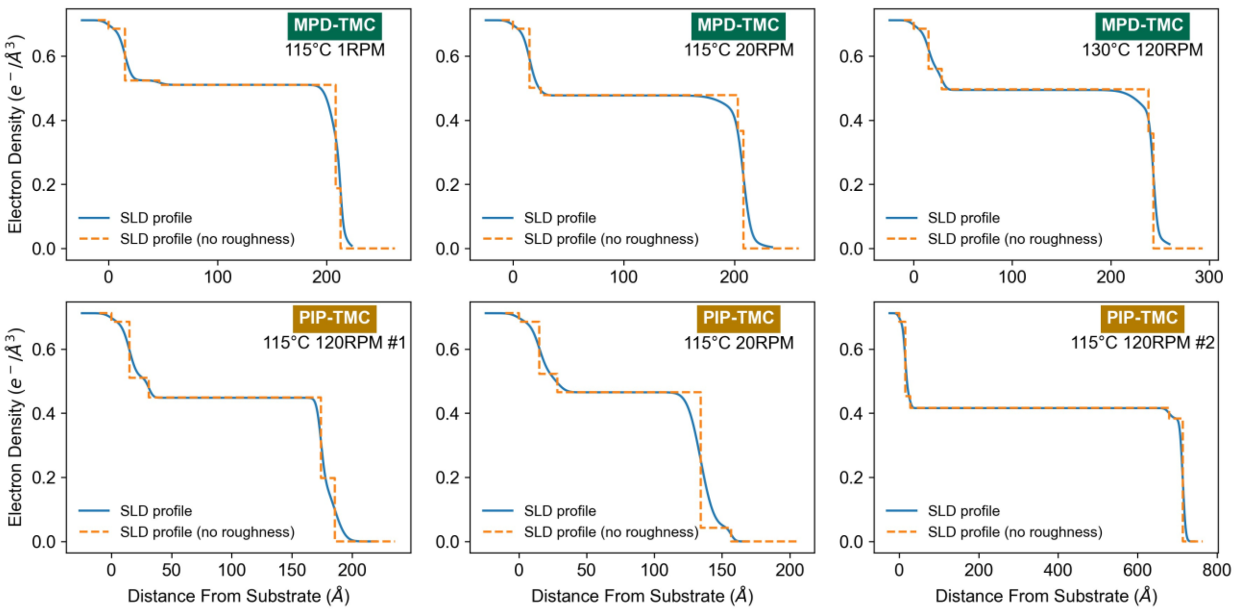

Figure S4. *Scattering length density (SLD) model profiles used to generate XRR model curves for each sample. The profile without roughness is shown as an aid for visualization of strata within the sample.*

F. Surface Functional Group Spacing

The bulk mass density of the film (ρ_M) and the GPC (growth per cycle) can be used to estimate the mass deposition rate, GPC_{mass} (ng·nm^{-2}·cycle^{-1}), the average number of amide bonds per volume (ρ_N, nm^{-3}), and the average number of amide bonds deposited per MLD cycle (ρ_A, nm^{-2}·cycle^{-1}). The bulk density is used rather than the top layer density because it represents the amount of material that propagates each MLD cycle.

$$GPC_{mass} = GPC \cdot \rho_M \quad (1)$$

$$\rho_N = 6\, M_{RU}^{-1} \cdot N_A \cdot \rho_M \quad (2)$$

$$\rho_A = \rho_N \cdot GPC \quad (3)$$

Here, M_{RU} is the molecular weight of a fully crosslinked repeat unit which contains six amide bonds (Figure 6). N_A is Avagadro's constant. The inverse of ρ_A, defined as $\bar{A}_{Amide}$, represents the average area surrounding a single amide bond. Area density is used here as it is reasonable to assume that polymerization occurs at a flat, substrate surface plane at each MLD step since growth per cycle is on the order of the size of a polyamide monomer.

$$\bar{A}_{Amide} = \rho_A^{-1} \quad (4)$$

To approximate the spacing between the amide bonds formed at each MLD cycle, a hexagonal array was used as shown in Figure S5. The array is used as a simplification, not a suggestion of crystallinity. This arrangement assumes that any segmental motion of polymer tails (in the loose, top stratum) is anchored to the points of the array as constrained by the crosslinked bulk layer. By equating $\bar{A}_{Amide}$ to the unit area of the array containing an equivalent of one amide bond, the average distance between each amide bond, d, may be calculated.

$$\bar{A}_{Amide} = \frac{\sqrt{3}}{2} d^2 \qquad (5)$$

$$d = \sqrt{\frac{2\sqrt{3}}{3 \cdot \rho_A}} \qquad (6)$$

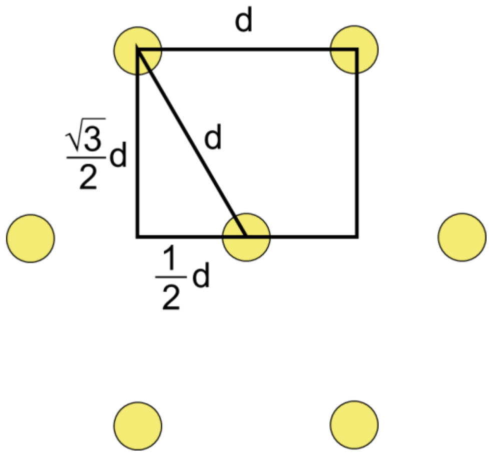

Figure S5. *Unit area geometry for a 2D hexagonal array, used as an approximate layout for functional end groups during MLD.*

G. X-ray Scattering Plots

In addition to the as-received commercial IP membranes and the MLD films presented in the main text, we also investigated the effect of rinsing the membranes (for preservative removal) on the structure of the commercial membranes. After rinsing the commercial MPD-TMC membrane, a small shift of the scattering feature at ~1.3 Å^{-1} towards higher q was observed (Figure S6a), corresponding to a decrease in the molecular packing distances and giving rise to this peak. Interestingly, the change in the q position of this

feature (proposed to be due to post-processing) is subtle compared to the shift to higher q observed for scattering features associated with small packing distances (>1.3 Å^{-1}). As shown in Figure S6b, minimal changes are observed in the PIP-TMC commercial membranes following rinsing.

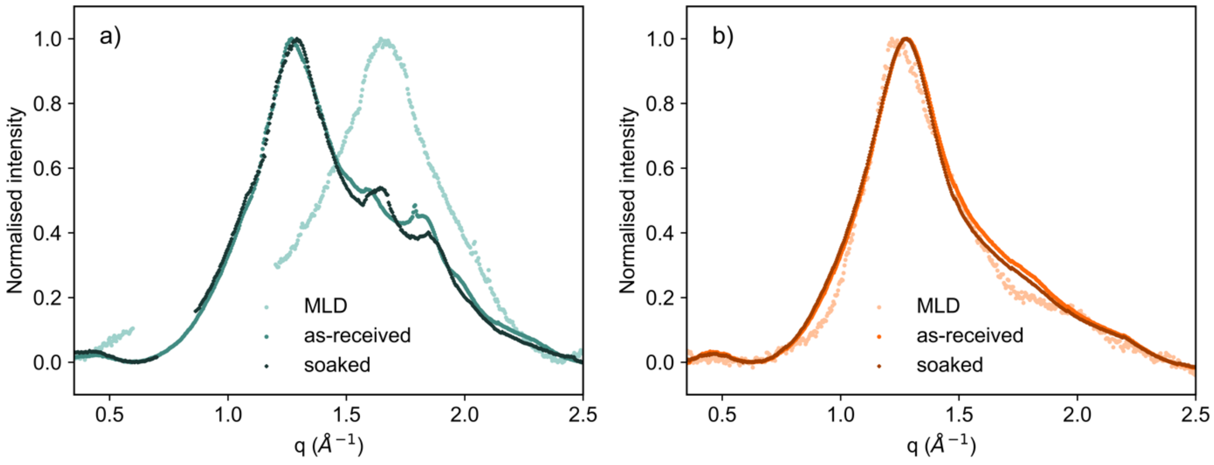

Figure S6. *1D integrated radial profile of the as-received and rinsed commercial IP membranes compared to films prepared by MLD for a) MPD-TMC and b) PIP-TMC chemistries. These data are normalized to the peak maximum between 1.0–2.0 Å^{-1} for easy visualization of changes in peak position and shape.*

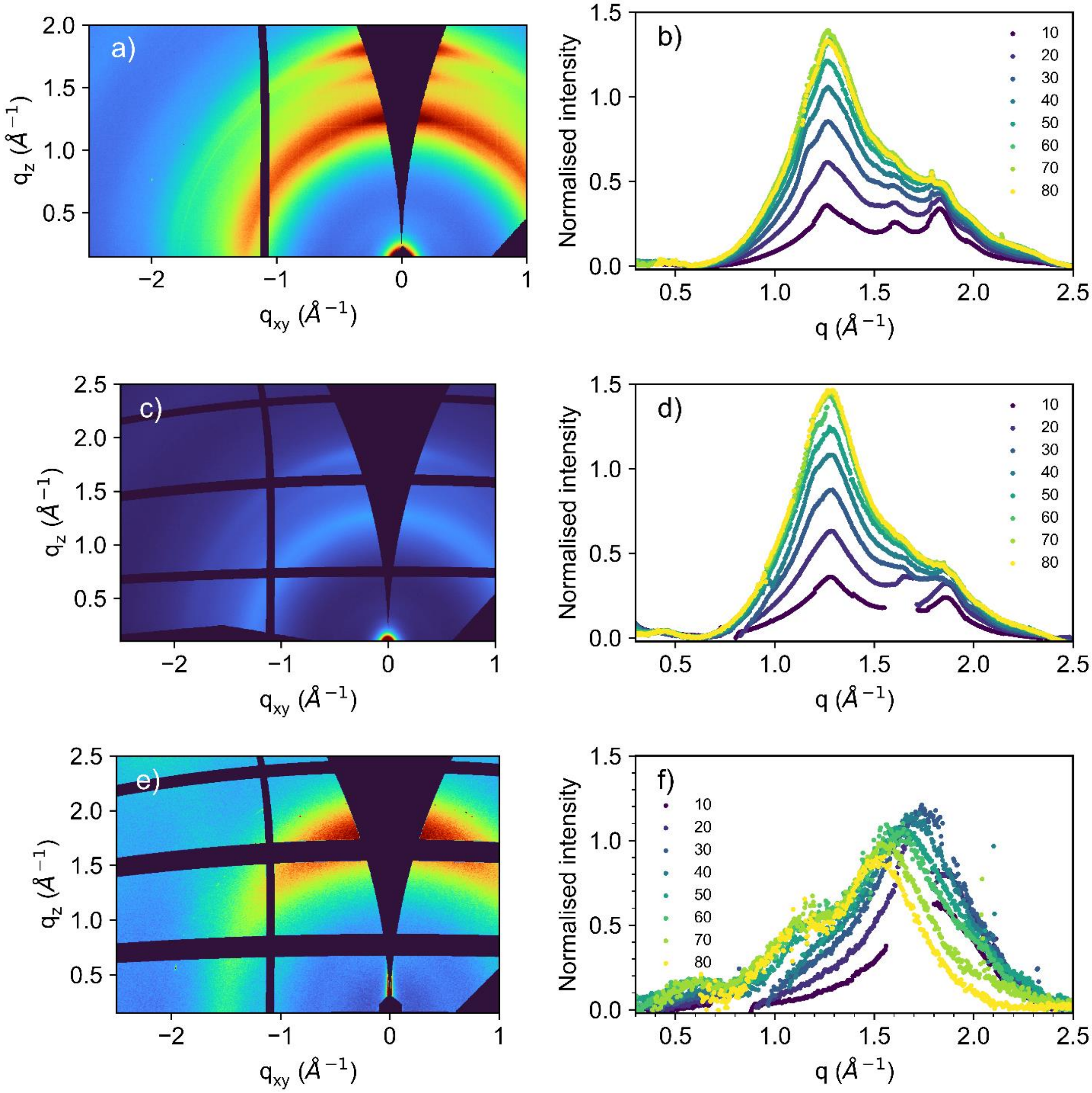

Figure S7. *(a), (c), and (e) q_{xy} vs q_z scattering plots and (b), (d), and (f) variations of scattering in chi over angles of 5–85°, taken in 10° slices for MPD-TMC films. (a) and (b) are measurements of the as-received commercial IP MPD-TMC TFC membrane (FilmTec XLE). (c) and (d) are measurements of the rinsed commercial IP MPD-TMC TFC membrane. (e) and (f) are measurements of a MLD MPD-TMC 180 nm film prepared at 115ºC. These data were intensity-scaled by $sin|chi|$, background-subtracted, and normalized to the peak maximum (between 1.0–2.0 Å^{-1}) when integrated over all angles. Note for (c) and (e), that single images are shown and analyzed due to difficulties in combining two images to account for the gaps in the detector.*

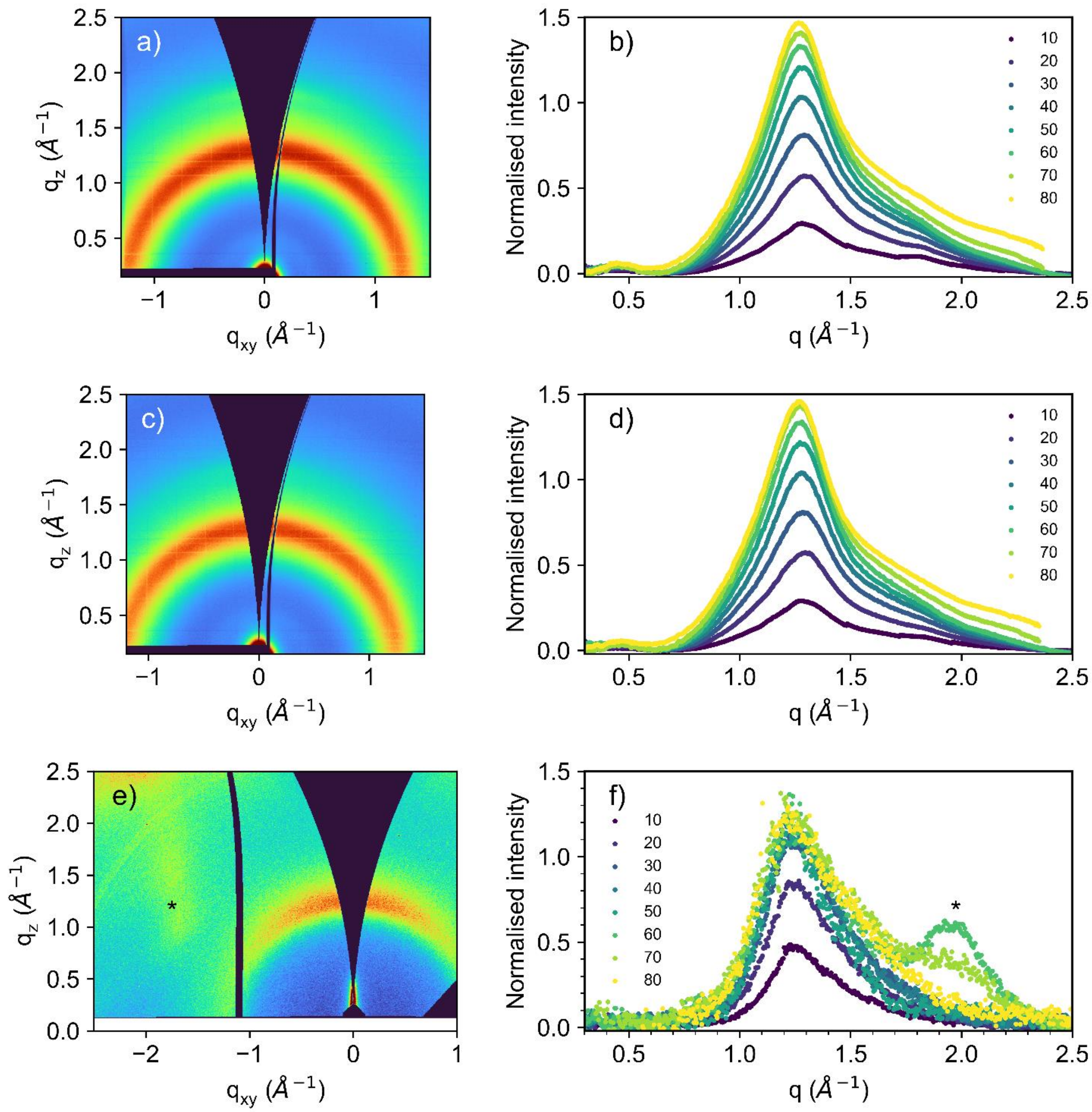

Figure S8. *(a), (c), and (e) q_{xy} vs q_z scattering plots and (b), (d), and (f) variations of scattering in chi over angles of 5–85°, taken in 10° slices for PIP-TMC films. (a) and (b) are measurements of the as-received commercial IP PIP-TMC TFC membrane (FilmTec NF270). (c) and (d) are measurements of the rinsed commercial IP PIP-TMC TFC membrane. (e) and (f) are measurements of the MLD PIP-TMC 64 nm film prepared at 115ºC. These data were intensity-scaled by $sin|chi|$, background-subtracted, and normalized to the peak maximum (between 1.0–2.0 $Å^{-1}$) when integrated over all angles. * highlights a scattering artifact in both the 2D and 1D data.*

References

(1) Zimudzi, T. J.; Sheffield, S. E.; Feldman, K. E.; Beaucage, P. A.; DeLongchamp, D. M.; Kushner, D. I.; Stafford, C. M.; Hickner, M. A. Orientation of Thin Polyamide Layer-by-Layer Films on Non-Porous Substrates. *Macromolecules* **2021**, *54* (24), 11296–11303. https://doi.org/10.1021/acs.macromol.1c02109.

(2) Larkin, P. J. Chapter 6 - IR and Raman Spectra–Structure Correlations: Characteristic Group Frequencies. In *Infrared and Raman Spectroscopy (Second Edition)*; Larkin, P. J., Ed.; Elsevier, 2018; pp 85–134. https://doi.org/10.1016/B978-0-12-804162-8.00006-9.
